# Role of rhinomanometry in the prediction of therapeutic positive airway pressure for obstructive sleep apnea

**DOI:** 10.1186/s12931-020-01382-4

**Published:** 2020-05-13

**Authors:** Yen-Bin Hsu, Stanley Yung-Chuan Liu, Ming-Ying Lan, Yun-Chen Huang, I-Shiang Tzeng, Ming-Chin Lan

**Affiliations:** 1 Department of Otolaryngology-Head & Neck Surgery, Taipei Veterans General Hospital, Taipei, Taiwan; 2 School of Medicine, National Yang-Ming University, Taipei, Taiwan; 3 Division of Sleep Surgery, Department of Otolaryngology, Stanford University School of Medicine, Stanford, CA USA; 4 Department of Otolaryngology-Head & Neck Surgery, Taipei Tzu Chi Hospital, Buddhist Tzu Chi Medical Foundation, New Taipei City, Taiwan; 5 School of Medicine, Tzu Chi University, Hualien, Taiwan; 6 Department of Research, Taipei Tzu Chi Hospital, Buddhist Tzu Chi Medical Foundation, New Taipei City, Taiwan

**Keywords:** Nasal resistance, Obstructive sleep apnea, Positive airway pressure, CPAP, Rhinomanometry

## Abstract

**Background:**

This study was conducted to evaluate the relationship between nasal resistance in different posture and optimal positive airway pressure (PAP) level. Other potential factors were also assessed for possible influence on PAP pressure.

**Methods:**

Forty- three patients diagnosed with obstructive sleep apnea (OSA) were prospectively recruited in this study. Nasal resistance was assessed by active anterior rhinomanometry in a seated position and then in a supine position at pressures of 75, 150, and 300 pascal. The factors correlating with PAP pressure were analyzed, including nasal resistance and patients’ clinical data.

**Results:**

Univariate analysis revealed that PAP pressure was correlated to nasal resistance in the supine position at 75 and 150 pascal (SupineNR75 and SupineNR150) (*P* = 0.019 and *P* = 0.004 in Spearman’s correlation coefficient analysis), but not correlated to nasal resistance in the seated position at different pressures or in the supine position at 300 pascal. The multiple linear regression analysis revealed that both SupineNR150 and body mass index (BMI) significantly predicted PAP pressure (β = 0.308, *p* = 0.044; β = 0.727, *p* = 0.006). The final PAP pressure predictive model was:

PAP pressure = 0.29 BMI + 2.65 SupineNR150 + 2.11.

**Conclusions:**

Nasal resistance in the supine position measured at 150 pascal may provide valuable information regarding optimal PAP pressure. Rhinomanometry should be included in the treatment algorithm of OSA patients when PAP therapy is considered.

## Backgrounds

Positive airway pressure (PAP) device is generally considered the first-line treatment for moderate to severe obstructive sleep apnea (OSA) [1]. PAP device serves as a “pneumatic splint” to overcome upper airway collapsibility via positive pressure from a tightly sealed nasal or oral mask. Randomized controlled trials have shown benefits of PAP device, which include improvements in daytime sleepiness, cognitive performance, blood pressure, and overall quality of life [2–6]. However, the efficacy of PAP therapy is limited by poor acceptance and compliance. It has been reported that rates of compliance approximate 50% [7]. An often cited reason for PAP intolerance refers to nasal problems, which accounts for 30–50% of cases [8]. These side effects include nasal congestion, postnasal drip, crusting, mucosal dryness, and recurrent sinusitis [9, 10].

Optimal PAP titration is an important issue. There are several methods to determine optimal PAP titration: (1) conventional polysomnography (PSG) in the sleep laboratory, which is time consuming and expensive; (2) split night PSG, which combines diagnostic PSG in the first half of night and therapeutic PSG in the second half of night; (3) automatic positive airway pressure (APAP), which determines optimal pressure level by individual device algorithms; and (4) mathematical formulas comprised of PSG parameters and anthropometric variables in prediction models [11].

The mathematical formulas which predict optimal pressure may reduce time and cost at the sleep lab during initial PAP titration studies. Previous studies have demonstrated that PSG parameters [apnea-hypopnea index (AHI), oxygen desaturation index (ODI) and mean oxygen saturation] and anthropometric variables [body mass index (BMI), neck circumference, Friedman’s tongue position and hyoid-mental distance] were established variables in a mathematical model to predict PAP pressure [12, 13].

Although the nasal airway contributes more than 50% of upper airway resistance, few studies have incorporated nasal resistance as a potential predictor to evaluate optimal PAP pressure [11]. Currently, nasal resistance is thought to be a contributing factor for snoring and mild OSA, but does not play a major role in moderate to severe OSA [14, 15].

In this study, rhinomanometry was used to assess the degree of nasal resistance. Nasal resistance was measured in the seated and supine position at different pressures. The aim of this study was to investigate the influence of nasal resistance in different positions on optimal PAP pressure.

## Methods

### Subjects and procedures

From January 2018 to March 2019, 43 subjects aged between 20 to 70 years were recruited prospectively. All subjects were diagnosed with OSA if they had AHI ≥5 episodes per hour of sleep and OSA-related symptoms. Exclusion criteria included smoking, previous nasal surgery, and diagnosis of nasal polyp or tumor. Subjects were instructed not to use any oral or topical nasal medications one week before rhinomanometric measurement. Subjects underwent comprehensive examination, including anterior rhinoscopy, nasopharyngoscopy, active anterior rhinomanometry, polysomnography and continuous positive airway pressure (CPAP) titration at the sleep lab. Modified Mallampati score and Epworth Sleepiness Scale were obtained. This study was approved by the Taipei Tzu Chi Hospital Institutional Review Board (no. 05-X06–014).

### Rhinomanometry

Subjects were instructed to rest in a quiet room with temperature control to maintain a relaxed state for 5 min [16]. Nasal resistance was assessed by active anterior rhinomanometry. All rhinomanometric measurements were performed using the NR 6-rhinomanomer (GM Instruments Ltd., Glasgow, UK) at pressures of 75, 150, and 300 pascal. The assessment was first in the seated position and then in the supine position. All the tests were finished in 25 min to avoid the possible effects from nasal cycle.

### Polysomnography

Standard PSG was performed in all subjects by trained sleep technicians. Electroencephalography, electrooculography, electrocardiography and electromyography for submentalis and tibialis anterior muscle were monitored continuously. Oronasal airflow was measured by thermistor. Thoraco-abdominal movements were recorded by piezoelectric bands. Apnea events were classified into obstructive apnea, central apnea and mixed apnea. An obstructive apnea event was defined as an absence or greater than 90% reduction in baseline airflow for at least 10 s with continued respiratory effort detected by the chest and abdomen movement channels. A hypopnea event was defined as greater than 30% reduction in the baseline airflow for at least 10 s and associated with 3% oxygen desaturation or an electroencephalogram arousal. AHI represented the total number of apnea and hypopnea events per hour of sleep.

### Optimal PAP titration

All subjects underwent nasal CPAP titration. All procedures were performed based on the criteria described in the American Academy of Sleep Medicine (AASM) clinical guidelines. The nasal masks were fitted by trained sleep technicians during the period of calibration. An optimal titration indicates that AHI is less than 5 events per hour for at least a 15-min duration, and supine REM sleep at the selected pressure is not continually interrupted by spontaneous arousal or awakenings [17].

### Statistical analysis

All statistical analysis was carried out using SPSS version 20.0 (IBM Corp., Armonk, NY). Continuous data was expressed as mean ± standard deviation, while categorical data was expressed as numbers and percentages. Spearman’s rank correlation coefficient was utilized to evaluate the correlation between PAP pressure and nasal resistance in the seated position and in the supine position at different pressures. A linear regression model was developed to identify significant contributors to optimal PAP pressure. Differences between groups were compared using Mann-Whitney U test for nonparametric continuous data. P value less than 0.05 was considered statistically significant.

## Results

Of the 43 OSA subjects with complete records, there were 32 males (74.4%) and 11 females (25.6%). The mean age was 48.05 years (range, 25–68 years), and the mean BMI was 27.81 kg/m^2^ (range, 19.40–41.00 kg/m^2^). Demographic data is shown in Table 1. AHI ranged from 6.90 to 93.20 events per hour, with a mean of 43.04 events per hour. ODI ranged from 4.90 to 89.90 events per hour, with a mean of 36.43 events per hour. PAP pressure ranged from 6 to 12 cm of water pressure (cwp), with a mean of 8.42 ± 1.8 cwp.

**Table 1:** Patient characteristics

Variables	Median	Range
Males (n, %)	32 (74.4%)	–
Females (n, %)	11 (25.6%)	–
Age, years	49.00	25–68
BMI, kg/m^2^	26.80	19.40–41.00
ODI, events/hr	34.40	4.90–89.90
AHI, events/hr	40.50	6.90–93.20
PAP pressure	8	6–12
Neck circumference, cm	39	30–46
ESS	10	2–22
Tonsil size	1	0–2
MMS	3	2–4

*BMI* body mass index, *ODI* oxygen desaturation index, *AHI* apnea-hypopnea index, *PAP* positive airway pressure, *ESS* Epworth Sleepiness Scale, *MMS* Modified Mallampati score

Spearman’s correlation coefficient analysis showed strong positive correlations between PAP pressure and BMI (r = 0.73, *P* < 0.001) and AHI (r = 0.62, *P* < 0.001) (Table 2). Moderate positive correlation was noted between PAP pressure and nasal resistance in the supine position at 150 pascal (SupineNR150; r = 0.49, *P* = 0.004) and age (r = − 0.458, *P* = 0.002) and neck circumference (r = 0.553, *P* < 0.001). Weak positive agreement was noted between PAP pressure and nasal resistance in the supine position at 75 pascal (SupineNR75; r = 0.37, *P* = 0.019). No significant correlations were found between PAP pressure and nasal resistance in the seated position at 75, 150 and 300 pascal (SeatedNR75, SeatedNR150, and SeatedNR300).

**Table 2:** Spearman’s correlation between positive airway pressure level and collected variables

Variables	Coefficient	*P* Value
Age, years	−0.46	0.002
BMI, kg/m^2^	0.73	< 0.001
AHI, events/hr	0.62	< 0.001
ESS	0.15	0.342
Neck circumference, cm	0.55	< 0.001
Tonsil size	0.28	0.070
MMS	0.19	0.239
SeatedNR75	0.06	0.711
SeatedNR150	−0.19	0.265
SeatedNR300	−0.07	0.733
SupineNR75	0.37	0.019
SupineNR150	0.49	0.004
SupineNR300	0.42	0.057

*BMI* body mass index, *AHI* apnea-hypopnea index, *ESS* Epworth Sleepiness Scale, *MMS* Modified Mallampati score, *SeatedNR75*, *SeatedNR150*, *SeatedNR300* nasal resistance in the seated position at 75, 150 and 300 pascal, *SupineNR75*, *SupineNR150*, *SupineNR300* nasal resistance in the supine position at 75, 150 and 300 pascal

A multiple linear regression analysis was used to test if PSG parameters, anthropometric factors and nasal resistance could predict optimal PAP pressure. SupineNR150 significantly predicted optimal PAP pressure (β = 0.308, *p* = 0.044), as did BMI (β = 0.727, *p* = 0.006). The results of the regression indicated two predictors accounting for 70.5% of the variance [R2 = 0.705, F (8,23) = 6.86, *p* < 0.001].

The final PAP pressure predictive model was:

$$ \mathrm{PAP}\ \mathrm{pressure}=0.29\ \mathrm{BMI}+2.65\ \mathrm{SupineNR}150+2.11 $$

This model indicated that optimal PAP pressure is significantly correlated with BMI and SupineNR150 (Table 3).

**Table 3:** Multiple linear regression analysis to predict the potential variables for positive airway pressure level.

Variables	B	β	95% CI	*P* Value
Sex	−0.74	− 0.20	−2.11 – 0.64	0.280
Age, years	0.002	0.01	− 0.04 – 0.05	0.927
BMI, kg/m2	0.29	0.73	0.09–0.48	0.006
AHI, events/hr	0.01	0.17	−0.01 – 0.03	0.235
Neck circumference, cm	−0.06	−0.11	− 0.33 – 0.21	0.639
Tonsil size	−0.23	−0.05	−1.54 – 1.09	0.726
MMS	0.11	0.04	−0.68 – 0.90	0.774
SupineNR75	1.52	0.19	−0.49 – 3.52	0.133
SupineNR150	2.65	0.31	0.003–5.29	0.044

*BMI* body mass index, *AHI* apnea-hypopnea index, *MMS* Modified Mallampati score, *SupineNR75*, *SupineNR150* nasal resistance in the supine position at 75 and 150 pascal

**Fig. 1:**
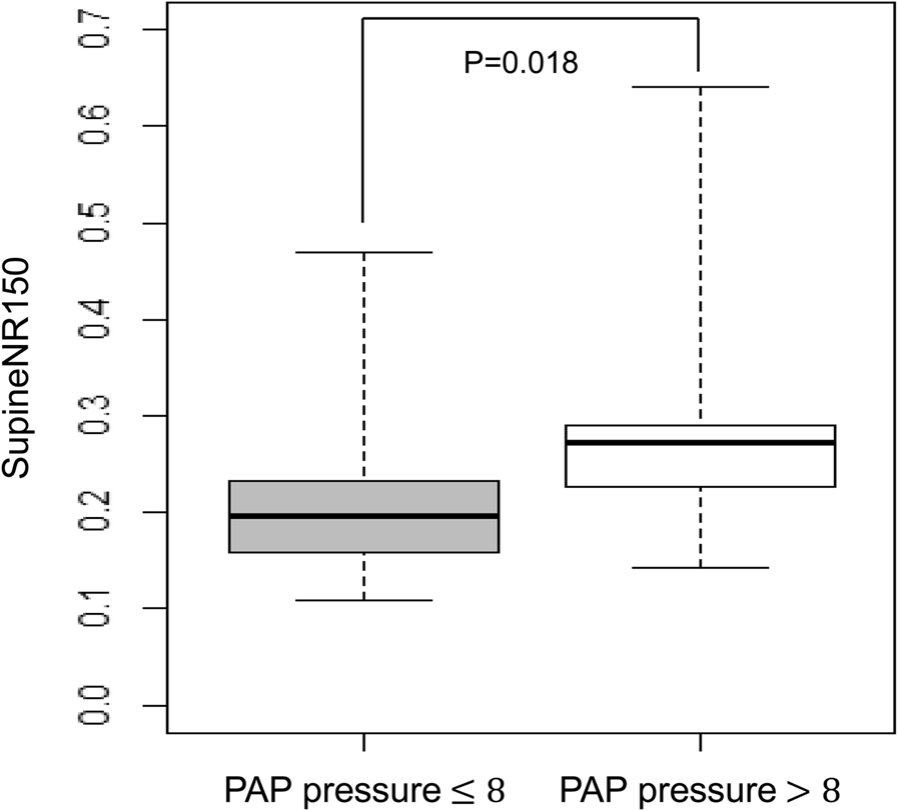
Patients with PAP pressure > 8 were associated with a significantly higher SupineNR150 compared to patients with PAP pressure ≤ 8. PAP, positive airway pressure; SupineNR 150, nasal resistance in the supine position at 150 pascal

Subjects with PAP pressure greater than 8 cwp were associated with a significantly higher SupineNR150 (*P* = 0.018) compared to patients with PAP pressure less than or equal to 8 cwp. No significant associations were found between nasal resistance in the seated position at 75, 150 and 300 pascal (SeatedNR75, SeatedNR150, SeatedNR300) and different degrees of PAP pressure (PAP pressure ≤ 8 or > 8 cwp). (Table 4 and Fig. 1).

**Table 4:** Associations between nasal resistance and positive airway pressure level.

Nasal Resistance		Mean ± SD	*P* Value
SeatedNR75	PAP pressure ≤ 8	0.155 ± 0.459	0.970
	PAP pressure > 8	0.162 ± 0.023	
SeatedNR150	PAP pressure ≤ 8	0.215 ± 0.061	0.165
	PAP pressure > 8	0.217 ± 0.028	
SeatedNR300	PAP pressure ≤ 8	0.307 ± 0.084	0.467
	PAP pressure > 8	0.295 ± 0.036	
SupineNR75	PAP pressure ≤ 8	0.170 ± 0.074	0.060
	PAP pressure > 8	0.211 ± 0.043	
SupineNR150	PAP pressure ≤ 8	0.224 ± 0.091	0.018^a^
	PAP pressure > 8	0.281 ± 0.055	
SupineNR300	PAP pressure ≤ 8	0.313 ± 0.116	0.140
	PAP pressure > 8	0.379 ± 0.075	

*PAP* positive airway pressure, *SeatedNR75*, *SeatedNR150*, *SeatedNR300* nasal resistance in the seated position at 75, 150 and 300 pascal, *SupineNR75*, *SupineNR150*, *SupineNR300* nasal resistance in the supine position at 75, 150 and 300 pascal

^a^ Indicates statistical significance

## Discussion

Conventional PSG with manual titration of optimal PAP pressure is considered the gold standard method for starting PAP therapy. However, it can be costly and time-consuming. Researchers have long been exploring for reliable mathematic models to predict optimal therapeutic PAP pressure settings. Several potential predictors to formulate mathematic models, which include BMI, neck circumference, AHI, ODI or mean oxygen saturation, were previously explored [12, 13]. However, nasal resistance has rarely been examined as a potential variable to estimate optimal therapeutic PAP pressure setting. This study aimed to explore the relationship between nasal resistance in different posture and optimal PAP pressure.

Previous studies have reported that subjective perception of nasal obstruction was poorly correlated with actual nasal resistance [18–20]. Active anterior rhinomanometry, which assesses nasal pressure and airflow during respiration, is able to determine the nasal resistance objectively. It is good for assessing the presence and severity of obstruction [21]. Although there are some shortcomings of this technique, including incapability to detect obstructive area precisely and the need of specialized equipment and a well-trained operator, active anterior rhinomanometry is still the most commonly used method and also considered the gold standard for evaluation of nasal resistance [22, 23].

In this study, multiple linear regression analysis indicated that BMI and SupineNR150 were contributing factors to PAP pressure. The influence of nasal resistance was less than that of BMI (β = 0.308, *p* = 0.044 vs β = 0.727, *p* = 0.006). Hueto et al. had previously shown that BMI and nasal resistance in the supine position after vasoconstriction at rhinomanometry pressure of 150 pascal were significant predictors of pressure settings [11]. However, the nasal resistance was measured after vasoconstriction in their study, whereas we measured nasal resistance without the use of a decongestant. Topical nasal decongestants raise vasoconstrictor tone which in turn increases nasal patency and reduces nasal resistance. Our study design approximates a more native physiologic environment.

Although nasal resistance is not considered a significant contributor in patients with moderate to severe OSA, it does play an important role in CPAP treatment. Nakata et al. have indicated that nasal resistance was higher in nasal CPAP failure patients compared to CPAP compliant patients, and the PAP titration level decreased significantly after nasal surgery [24]. Camacho et al. concluded in the systematic review and meta-analysis that nasal surgery not only decreased therapeutic CPAP pressure but also increased CPAP compliance [25]. Sugiura et al. have shown that nasal resistance and AHI were statistically significant variables to predict acceptance of CPAP, and a higher nasal resistance might result in CPAP non-acceptance [26]. Morris et al. have found that nasal cross-sectional area at the head of inferior turbinate differed significantly between CPAP-tolerant and CPAP-intolerant patients, supporting a major role of nasal resistance in CPAP therapies [18]. Powell et al. have demonstrated that radiofrequency reduction of turbinate hypertrophy significantly increased adherence to CPAP [27].

Of interest to our study design which included assessment of nasal resistance in the supine position, Tarrega et al. previously reported that nasal resistance assessed by active anterior rhinomanometry correlated poorly with PAP treatment levels [28]. Nonetheless, they measured nasal resistance only in the seated position, which could not represent the natural sleep position. The measurements might not accurately reflect genuine nasal condition during sleep. In this study, we also found no significant correlations between PAP pressure and nasal resistance in the seated position at different pressures. However, we noted that nasal resistance in the supine position measured at 150 pascal was a significant predictor of therapeutic PAP pressure.

There are several limitations in this study. First, the study population is mainly comprised of patients with moderate to severe OSA because CPAP is considered as first-line therapy for patients with moderate to severe OSA. Therefore, the results may not fully represent the general OSA population. Second, the influence of nasal cycle may result in side to side variation of nasal resistance over a period of hours. Nevertheless, the total nasal resistance remains rather stable despite side to side fluctuation of nasal resistance. In this study, we measured total nasal resistance to prevent the possible effect of nasal cycle. Third, the results of rhinomanometry measurements might be partially influenced by adaptations to environmental changes. The difference of nasal resistance between the seated and supine positions may result from better acclimation to the room atmosphere. Therefore, all subjects in this study were instructed to rest for at least 5 min in the examination room before being assessed by active anterior rhinomanometry to minimize the effect of acclimation. Finally, the study population is relatively small. Larger studies may be warranted to confirm the role of rhinomanometry in the prediction of therapeutic PAP pressure for OSA.

## Conclusions

Nasal resistance in the supine position at 150 pascal is a significant predictor for optimal PAP pressure. An objective assessment of nasal obstruction by rhinomanometry is warranted in OSA patients when considering CPAP treatment. Nasal resistance in the supine position may provide valuable information regarding optimal PAP pressure and further need for nasal surgery.

## Data Availability

All data generated or analyzed during the present study are included in this published article.

## Abbreviations

AASM: American Academy of Sleep Medicine
AHI: Apnea-hypopnea index
APAP: Automatic positive airway pressure
BMI: Body mass index
CPAP: Continuous positive airway pressure
cwp: Cm of water pressure
ODI: Oxygen desaturation index
OSA: Obstructive sleep apnea
PAP: Positive airway pressure
PSG: Polysomnography
SeatedNR75, SeatedNR150, SeatedNR300: Nasal resistance in the seated position at 75, 150 and 300 pascal
SupineNR75, SupineNR150, SupineNR300: Nasal resistance in the supine position at 75, 150 and 300 pascal
